# A Novel Strategy for Inducing Enhanced Mucosal HIV-1 Antibody Responses in an Anti-Inflammatory Environment

**DOI:** 10.1371/journal.pone.0015861

**Published:** 2011-01-06

**Authors:** Frank Wegmann, George Krashias, Kerstin Lühn, Karoliina Laamanen, Sueli Vieira, Simon A. Jeffs, Robin J. Shattock, Quentin J. Sattentau

**Affiliations:** 1 The Sir William Dunn School of Pathology, University of Oxford, Oxford, United Kingdom; 2 Weatherall Institute of Molecular Medicine, University of Oxford, Oxford, United Kingdom; 3 The Jefferiss Trust Research Laboratories, Imperial College Faculty of Medicine, London, United Kingdom; 4 Centre for Infection, St George's, University of London, London, United Kingdom; University of Toronto, Canada

## Abstract

Prophylactic vaccination against HIV-1 sexual transmission will probably require antibody elicitation at genital mucosal surfaces. However, HIV-1 envelope glycoprotein (Env)-based antigens are weakly immunogenic, particularly when applied mucosally. The polyanion PRO 2000 is safe for human vaginal application, and thus may represent a potential formulating agent for vaginal delivery of experimental vaccine immunogens. Based upon its biochemical properties, we hypothesized that PRO 2000 might enhance mucosal immunogenicity of HIV-1 envelope glycoprotein (Env)-based antigens, promoting local and systemic immune responses. Vaginal immunization with Env-PRO 2000 resulted in significantly increased titres of Env-specific mucosal IgA and IgG in mice and rabbits, respectively, compared to Env alone, revealing modest but significant mucosal adjuvant activity for PRO 2000. In vitro, PRO 2000 associated with Env, protecting the glycoprotein from proteolytic degradation in human vaginal lavage. Unexpectedly, PRO 2000 antagonized TLR4 activation, suppressing local production of inflammatory cytokines. Since inflammation-mediated recruitment of viral target cells is a major risk factor in HIV-1 transmission, the immune modulatory and anti-inflammatory activities of PRO 2000 combined with its intravaginal safety profile suggests promise as an HIV-1 mucosal vaccine formulating agent.

## Introduction

Despite increasing access to antiretroviral drugs in developing countries, prevention or reduction of HIV-1 sexual transmission is needed to contain the continuing growth of the pandemic [1]. One of the most effective preventive strategies against many infectious diseases is prophylactic vaccination. However, an efficaceous HIV-1 vaccine remains unavailable. A major element of HIV-1 vaccine design is the induction of neutralizing antibodies by immunization with recombinant HIV-1 envelope glycoproteins (Env) or engineered fragments thereof [2], [3]. Since HIV-1 is transmitted predominantly sexually, the most appropriate site to elicit an antibody barrier is at the genital mucous membranes [4], [5]. At present, we do not know how to induce long-term mucosal immunity against HIV-1 by conventional immunization strategies, and obtaining high antibody titres at mucosal surfaces appears to be regulated by mechanisms distinct from the systemic immune system [6], [7]. Thus the induction of long-lived mucosal immunity may require vaccine administration directly to the mucosae, especially if the efficient induction of antigen-specific IgA secretion is required [8]. However, HIV-1 Env-based antigens generally lack robust intrinsic immunogenicity, and there are no licensed mucosal adjuvants currently available. Moreover, caution must be exercised when considering the use of adjuvants in a mucosal context, since mucosal application of an adjuvant-containing formulation may induce local inflammation, potentially increasing the HIV-1 transmission risk by recruitment of activated CD4+ T cells that are the primary targets for HIV-1 replication in vivo [9], [10], [11]. Thus adjuvants for mucosal HIV-1 immunization would ideally promote immune responses whilst maintaining a non-inflammatory environment.

In the absence of a vaccine, another strategy currently under development to reduce HIV-1 transmission is the use of topical microbicides [12]. PRO 2000 is an anionic polymer that was under investigation as a candidate microbicide, but was recently demonstrated to be ineffective at preventing HIV-1 transmission [13]. However, PRO 2000 has an excellent safety record for vaginal application with no evidence for local toxicity or irritation [14], [15], [16] and has been demonstrated to suppress the generation of vaginal inflammatory mediators in women [17]. Moreover, being a gel PRO 2000 has a relatively long residency time in the vaginal tract [16]. For these reasons, PRO 2000 might be a useful formulating agent for vaginally-applied HIV-1 vaccine antigens. An additional point is that similar to other polyanions [18], [19] PRO 2000 reversibly binds viral HIV-1 gp120 [20], and therefore may interact with soluble recombinant Env-based candidate vaccine antigens, modifying their antigenicity. Polyanion binding to gp120 selectively and reversibly masks antigenic surfaces containing positive charges, including the V3 loop and the CD4-induced (CD4i)-surface [18], [19]. Most of the V3 loop is considered too variable to be helpful as a broadly-specific neutralization target [21] and CD4i epitopes are poorly accessible to antibody on the intact viral spike and hence are poor neutralizing antibody targets [22]. We therefore hypothesized that the formation of reversible gp140-PRO 2000 complexes in a vaccine formulation might improve the antigenicity of gp140 by re-directing immune responses towards more conserved neutralization-relevant surfaces, and might additionally act as a depot, increasing antigen residency time and thereby immunogenicity. Since basic amino acids form the cleavage sites of most proteases [23], we also hypothesized that complexing of Env with a polyanion might protect the glycoprotein from proteolytic digestion, further enhancing the residency time of intact antigen at the mucosal surfaces. This would be of particular importance for a glycoprotein such as HIV-1 gp120, in which many of the conserved neutralization epitopes are highly conformational and discontinuous [24], [25].

Here we present proof of principle that co-formulation of a recombinant trimeric gp140 derived from a clade B/C HIV-1 isolate with the polyanion PRO 2000 results in favourably modified antigenicity, increased immunogenicity, and unexpectedly, reduced mucosal inflammatory responses as a result of TLR4 antagonism. We thus conclude that PRO 2000 may be a useful formulation agent for vaginal vaccine delivery.

## Results

### Immunogenicity of vaginally-applied gp140-PRO 2000 complexes in mice

Trimeric forms of HIV-1 Env may be superior at inducing neutralizing antibody responses [26], [27], [28], and Clade C dominates the worldwide HIV-1 pandemic [29]. We therefore used a soluble trimeric form of HIV-1 Env, comprising the membrane external Env sequence (gp140) from clade B/C recombinant CCR5-tropic (R5) Chinese viral isolate 96CN54, as antigen, here termed gp140_CN54_. This antigen was produced to good manufacturing practice standards, and contained undetectable levels of endotoxin contamination. Antigenicity and trimericity were assessed by surface plasmon resonance (SPR)-analysis and blue native PAGE, respectively. The antigen bound several conformation-dependent ligands including soluble CD4 (sCD4) and the neutralizing monoclonal antibody (NmAb) IgG1b12 [30], [31] (b12), and mAb binding to the CD4-induced (CD4i) surface was increased after sCD4 binding (Figure S1). Taken together these data suggest that the antigen adopts a properly-folded conformation with presentation of at least one well-conserved neutralizing epitope.

**Figure 1:**
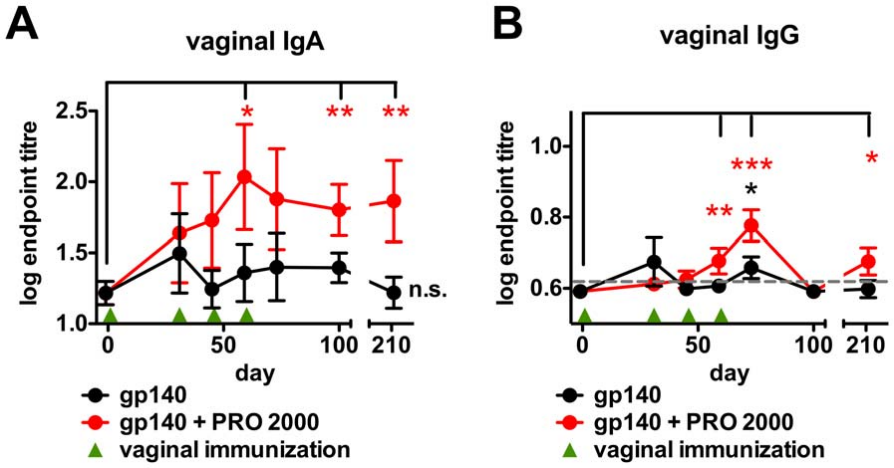
PRO 2000 co-application induces increased mucosal antibody responses in mice. BALB/c mice (n = 7) were immunized four times vaginally with 12.5 µg gp140 +/− 1% PRO 2000; Time courses of the vaginal lavage IgA (**A**) and IgG (**B**) endpoint titres. *gp140 + PRO* indicates combined gp140 and PRO 2000. Titre data were log-transformed and are shown as mean ± SEM. The grey dashed line indicates the ELISA detection limit. * *p*<0.05; ** *p*<0.01.

The vagina is generally considered to be poor site for immune induction [32], suggesting that the administration of an antigen in the absence of adjuvant is unlikely to elicit robust adaptive immune responses, and indeed may drive a tolerogenic response [33]. To test whether PRO 2000 modulated the immunogenicity of vaginally-applied Env antigen, two groups of mice were immunized intravaginally four times with 12.5 µg gp140_CN54_ in control gel alone, or in control gel supplemented with 1% PRO 2000. The PRO 2000 used here was confirmed to have <0.05 U/mL endotoxin. Analysis of vaginal lavage revealed that only the group receiving gp140 in PRO 2000 gel, but not antigen alone, elicited vaginal IgA responses significantly higher than the pre-immune samples (Figure 1A). The gp140-specific IgA responses elicited by administration of gp140 in PRO 2000 were stable between the time of the final boost at day 59 until the end of the experiment at day 210, implying unexpectedly good durability of these responses. At day 210, endpoint IgA titres were approximately 5-fold higher in the antigen + PRO 200 group than the group receiving antigen alone. Furthermore, when the two groups were directly compared by collectively analyzing all time points after the first immunization, a significant difference was found (*p* = 0.0286, Mann Whitney test). Vaginal IgG titres were significantly increased above baseline in the animals receiving antigen alone at only one time point, but were significantly increased at three time points in mice receiving gp140 in PRO 2000 (Figure 1B), although titres were relatively low and waned rapidly. When vaginal IgG titres of both groups were directly compared by collectively analyzing all time points after the first immunization no significant difference was found. Furthermore we were unable to detect any systemic responses in serum (data not shown). Taken together, these data indicate that PRO 2000 predominantly enhances compartmentalized mucosal IgA responses in mice.

### Immunogenicity of vaginally-applied gp140-PRO 2000 complexes in rabbits

**Figure 2:**
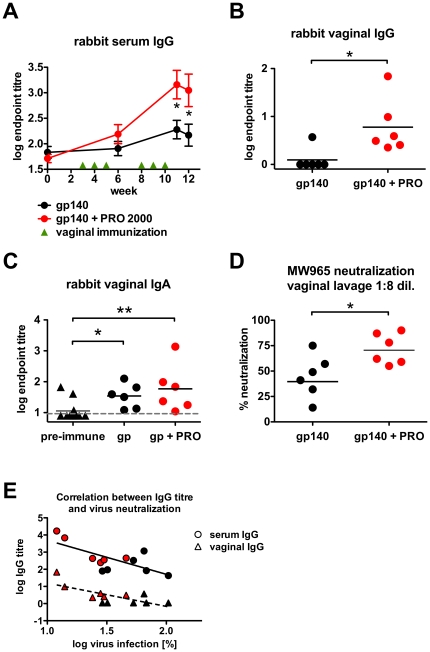
PRO 2000 co-application induces increased systemic and mucosal antibody responses in rabbits. New Zealand White rabbits (n = 6) received six vaginal immunizations using 50 mg antigen per dose in the presence or absence of 1% PRO 2000. (**A**) Time course of rabbit serum IgG endpoint titres. (**B**) Endpoint IgG titres of week 12 vaginal lavage samples. (**C**) Endpoint IgA titres of week 12 vaginal lavage samples. (**D**) MW965 pseudovirus neutralization of week 12 vaginal lavage samples. Horizontal bars represent geometric means. Samples were tested at 1∶8 dilution; 100% infection level was defined by pre-immune samples. (**E**) Correlation of the rabbit serum- and vaginal gp140-specific IgG responses with the MW965 pseudovirus neutralization (**D**) of week 12 samples. Red symbols represent data from animals immunized with gp140 + PRO 2000. *Pre-immune* indicates samples collected prior to the first immunization; *gp140 + PRO* indicates combined gp140 and PRO 2000. Titre data were log-transformed and are shown as mean ± SEM. The grey dashed line indicates the ELISA detection limit. * *p*<0.05; ** *p*<0.01.

To evaluate the immunomodulatory activity of PRO 2000 in a second species, we immunized rabbits intravaginally with antigen formulated in carrier gel alone or in PRO 2000 gel. We observed significant increases in systemic and vaginal IgG responses in the presence of PRO 2000 in rabbits after the second series of boosts (Figure 2A–B). Assay of vaginal lavage for antigen-specific IgA revealed increases compared to the pre-immune sera, but statistically equivalent titres in both gp140 alone and gp140-PRO 2000 groups, indicating that in this species PRO 2000 did not significantly increase IgA responses (Figure 2C). To test whether vaginal immunization induced neutralizing activity at the vaginal mucosa, week 12 rabbit vaginal lavage samples were analyzed in a pseudovirus neutralization assay. Significant neutralization of a sensitive tier 1 heterologous clade C pseudovirus (MW965) [34] was observed for both groups (Wilcoxon signed rank test *p* = 0.0313, respectively), and the gp140 + PRO 2000-immunized rabbits showed significantly increased neutralization compared to the gp140 alone group (Figure 2D, *p* = 0.0278, unpaired *t*-test). Limited yields of vaginal lavage unfortunately precluded further analysis of neutralization activity with *env* from other viral clones. The increased neutralization capacity might also be mediated by a PRO 2000-induced secretion of antiviral mediators or immunoglobulins, however a recent clinical study has shown that PRO 2000 dampened immune mediator secretion rather than inducing it [17]. The vaginal and systemic IgG titres correlated significantly with each other (Figure S2C) and with the neutralization capacity of the vaginal lavage samples (Figure 2E), whereas total vaginal immunoglobulin levels were indistinguishable between study groups (Figure S2A–B). This strongly suggests that neutralization was due to the local immunization-elicited antibody responses of the rabbits.

### Second-site adjuvantation of antigen + PRO 2000 formulations

**Figure 3:**
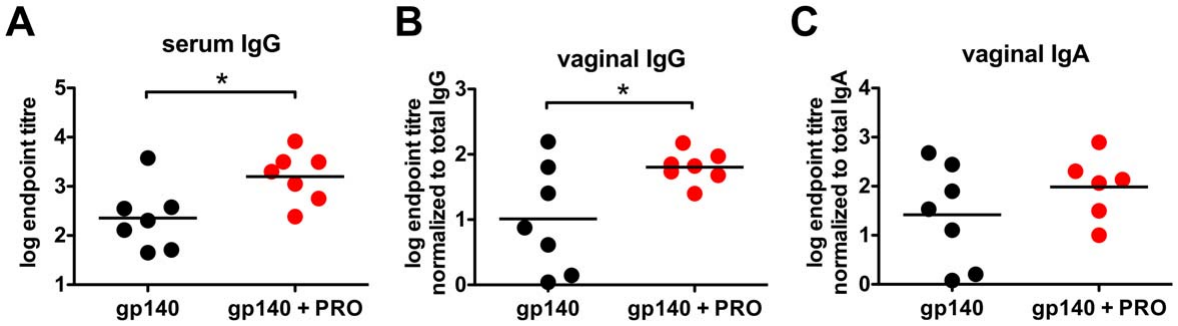
Second-site immunization in the presence of PRO 2000 increases systemic and mucosal antibody responses. BALB/c mice (n = 7) were primed intranasally with 10 µg gp140 per animal in polycation and were boosted three times vaginally with gp140 +/− PRO 2000. Samples were collected 14 days after the final vaginal immunization. Serum IgG endpoint titres (**A**), or vaginal lavage IgG (**B**) and IgA (**C**) endpoint titres normalized to the total IgG or IgA content or each sample, respectively, are displayed. *gp140 + PRO*, combination of gp140 and PRO 2000.* *p*<0.05.

To attempt to further increase immune responses to vaginal Env immunization, we combined the antigen-PRO 2000 delivery with an intranasal prime immunization, a route that is generally accepted as a good inducer of immunity at the vaginal mucosa [35], [36], [37]. As nasal immunizations are less likely to trigger genital mucosal inflammation and are therefore not associated with an increased risk of genital HIV-1 transmission, we used a more potent adjuvant for immunizations via the intranasal route. This mucosal priming adjuvant was a polycation-based agent (Figure 3) [38], [39] which has intrinsic adjuvant activity and induces a balanced Th response (our unpublished data). By contrast with the previous experiment, mice receiving the prime and three vaginal immunizations had readily detectable gp140-specific serum and vaginal IgG responses, which were significantly higher in the gp140 + PRO 2000-boosted group than the gp140 alone group (Figure 3A–B, *p* = 0.0195 and 0.0305 respectively, unpaired *t*-tests). Moreover, vaginal IgA responses were 3-fold increased in the gp140 + PRO 2000 group compared to gp140 alone (Figure 3C), although this trend did not reach significance. Thus mucosal priming at a second site with a heterologous adjuvant resulted in enhanced local and systemic antibody responses.

### PRO 2000 induces a T helper cell type-2 (Th2) immune bias

**Figure 4:**
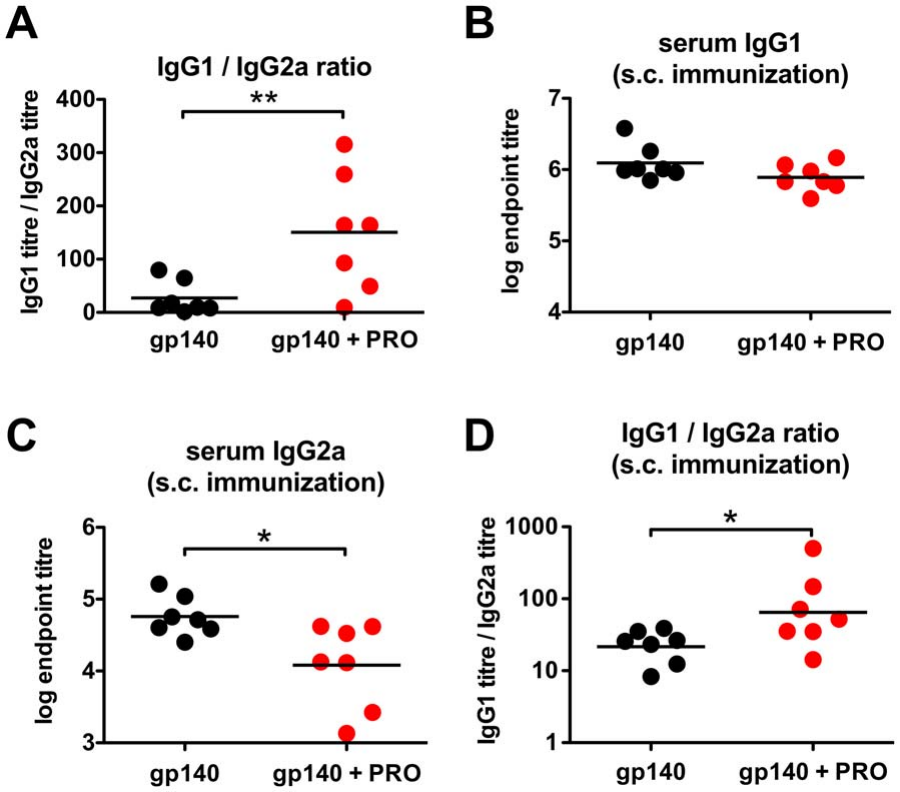
Influence of PRO 2000 co-administration on the type of T helper cell response. The serum specific IgG1- and IgG2a titres were determined as indicators of a Th2 or Th1 type of response, respectively. (**A**) Serum IgG1/IgG2a endpoint titre ratios of vaginally immunized mice (described in Figure 3A–C). (**B–D**) BALB/c mice (n = 7) were primed s.c. with gp140 +/− PRO 2000 in FCA and were boosted using the same route and formulation without FCA. (**B**) Serum IgG1 endpoint titres; (**C**) IgG2a endpoint titres; (**D**) IgG1/IgG2a endpoint titre ratio. *gp + PRO*, combination of gp140 and PRO 2000. * *p*<0.05; ** *p*<0.01.

**Figure 5:**
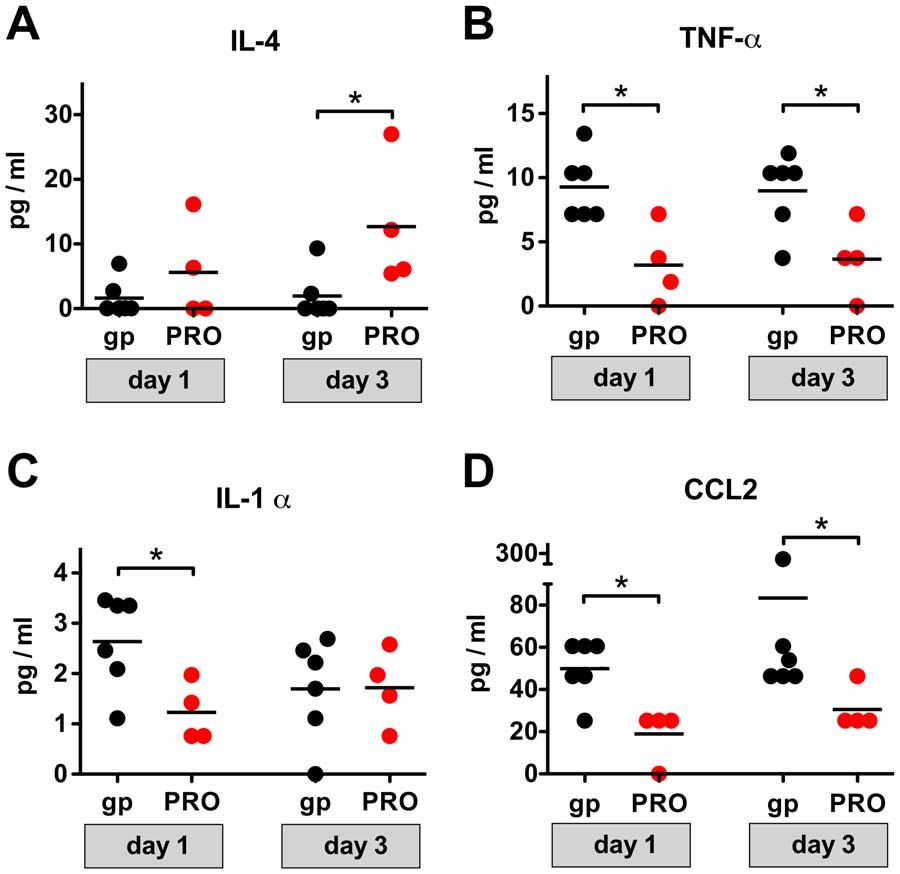
Immunomodulary activity of PRO 2000. Mononuclear cells derived from the vagina-draining Inguinal lymph nodes of vaginally immunized mice (described in Figure 3A–C) were cultured in the presence of 15 µg/ml of gp140, and the concentrations of cytokines/chemokines in supernatants were quantified on day 1 and 3 of the culture by multiplex assay. (**A**) IL-4, (**B**) TNF-α, (**C**) IL-1α, (**D**) CCL2. Results for individual mice are displayed. *gp*, gp140; *PRO*, combination of gp140 and PRO 2000. * *p*<0.05.

Recently it has been demonstrated that the recruitment of viral target cells, predominantly T helper type-1 (Th1)-activated CD4^+^ T cells, to the primary focus of mucosal HIV-1 replication is an important factor influencing the outcome of vaginal lentiviral infection [11]. Since both Th1 bias and T cell recruitment are primarily driven by a pro-inflammatory environment, we wished to determine whether PRO 2000 influenced the mucosal Th bias. The IgG1/IgG2a ratio is indicative of a Th2/Th1 adaptive immune bias. Analysis of the antigen-specific IgG1/IgG2a ratio in the serum samples reported on in Figure 3 revealed that mice boosted with the gp140 + PRO 2000 combination had a significantly greater IgG1/IgG2a ratio than those boosted with gp140 alone (Figure 4A *p* = 0.0055, Mann Whitney test), implying a Th2-biased response. To further investigate the Th2-biasing activity of PRO 2000, gp140 + PRO 2000 or gp140 alone were administered subcutaneously in Freund's Complete Adjuvant (FCA), a well-characterized and potent Th1-adjuvant [40]. Serum samples showed statistically indistinguishable gp140-specific IgG1 responses (Figure 4B), but significantly reduced IgG2a titres (Figure 4C, *p* = 0.0181, unpaired *t*-test) and significantly increased IgG1/IgG2a titre ratios (Figure 4D, *p* = 0.0379, Mann Whitney test) for animals receiving gp140 + PRO 2000 in FCA. Confirmation of a PRO 2000-induced local Th2 bias came from analysis of T cell cytokine profiles. Cells derived from the vagina-draining inguinal lymph nodes of vaginally-immunized mice (from the experiment shown in Figure 3) were cultured in gp140 and supernatants were analyzed by a multiplex cytokine/chemokine assay on days 1 and 3. Significantly increased levels of the Th2-cytokine IL-4 but decreased levels of pro-inflammatory cytokines TNF-α and IL-1 α were observed in mice immunized with gp140 + PRO 2000 (Figure 5A-C), diagnostic of a Th2 bias and demonstrating the anti-inflammatory activity of PRO 2000. Moreover, the chemokine CCL2 (MCP-1) was significantly decreased in cultures from gp140 + PRO 2000 immunized animals (Figure 5D). CCL2 has been shown to increase HIV-1 entry into resting CD4^+^ T cells [41] and trigger recruitment of HIV-1-susceptible target cells [42]. Thus reduced CCL2-secretion might defavour HIV-1 replication within the vaginal mucosa.

### PRO 2000 interferes with TLR4-signalling

**Figure 6:**
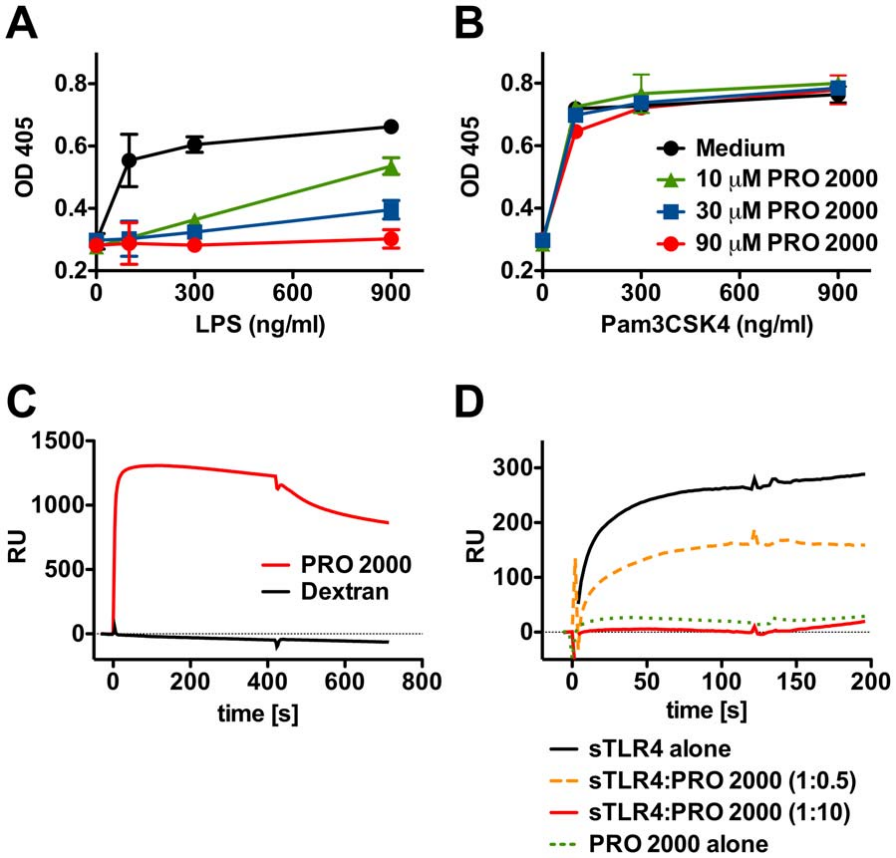
PRO 2000 inhibits LPS signalling and inhibits the TLR4-MD2-lipid A interaction. Murine embryonic fibroblast (MEF) TLR reporter cells were activated with ultra-pure LPS (**A**, corresponding legend in B) or with the synthetic TLR2 agonist Pam3CSK4 (**B**) in the presence of 10, 30 or 90 µM PRO 2000 or in medium alone. Means of triplicates ± SD are shown. For surface plasmon resonance binding assays (**C–D**), TLR4:MD2-complexes were immobilized on a CM5 chip (**C**), and binding of 100 µM PRO 2000 or low molecular weight dextran was observed, or lipid A was immobilized on a HPA chip (**D**) and binding of 155 nM TLR4:MD2, 1.54 µM or 77 nM PRO 2000 or combinations thereof were observed.

One mechanism for inducing a Th2-biased immune response might be for PRO 2000 to antagonize a default vaginal Th1 environment brought about by resident bacterial flora. PRO 2000 is structurally related to dextran sulfate, which has recently been shown to inhibit TLR4-induced maturation of dendritic cells by an unknown mechanism [43]. We therefore investigated whether PRO 2000 influenced the Th bias by interfering with TLR4-mediated signalling. We stimulated a murine TLR reporter cell line with ultra-pure *S. minnesota* LPS in the absence or presence of PRO 2000. LPS stimulation of the cells was blocked by PRO 2000 in a dose-dependent manner (Figure 6A), whereas Pam3CSK4 (a TLR2 agonist)-mediated stimulation of the same cell line was unaffected by PRO 2000 (Figure 6B). To evaluate whether PRO 2000 might directly bind TLR4 and block LPS binding, we used SPR to quantify PRO 2000 interactions with the LPS-binding complex of TLR4 and MD-2. PRO 2000 interacted strongly with immobilized human TLR4:MD-2 (Figure 6C). Since LPS interacts with TLR4:MD-2 via its lipid A moiety, we analyzed whether PRO 2000 bound specifically to the lipid A interaction site on the TLR4:MD-2-complex. Soluble TLR4:MD-2 with or without PRO 2000 were flowed over immobilized lipid A at different molar ratios. We confirmed previous observations that soluble TLR4:MD-2 directly interacts with immobilized lipid A, whereas PRO 2000 showed no detectable lipid A binding (Figure 6D). The lipid A-TLR4:MD-2 interaction was inhibited in a dose-dependent manner when PRO 2000 was co-injected, and was abolished by a 10-fold molar excess of PRO 2000. These data demonstrate that PRO 2000 binds TLR4:MD-2, thereby inhibiting LPS-engagement of the receptor.

### Antigenicity and stability of gp140-PRO 2000 complexes

**Figure 7:**
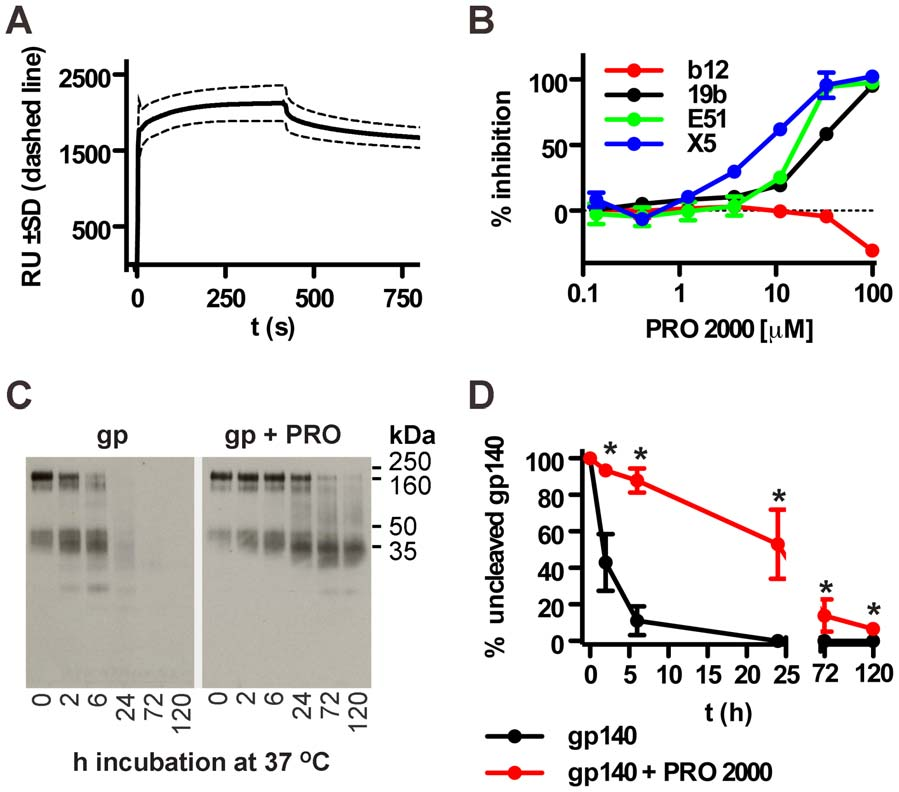
PRO 2000 binds the gp140_CN54_ coreceptor binding site and protects against vaginal lavage-mediated degradation. (**A**) gp140_CN54_ and the control protein BSA were immobilized on a CM5 sensor chip and 100 µM PRO 2000 was flowed over the chip. The mean curve (solid line) ± SD (dashed line) of three independent experiments is shown. (**B**) Competition of PRO 2000 with mAb-binding (mAbs used at 50% binding concentration) was determined by ELISA using immobilized gp140_CN54_; Means of triplicates ± SD are shown. (**C**) Western blot of gp140_CN54_ in human vaginal lavage incubated either alone or in the presence of 100 µM PRO 2000 at 37°C for the indicated periods of time. Uncleaved gp140 shows an apparent mol wt of 160 kDa. (**D**) Quantification of four independent stability experiments ± SD. *p<0.05.

A previous study has reported reversible interactions between HIV-1 gp120 derived from the MN and YU2 isolates and PRO 2000 [20]. Since such interactions may transiently alter antigenicity in vitro and potentially in vivo by masking certain antigenic surfaces, we tested whether PRO 2000 interacts with gp140_CN54_ using SPR. PRO 2000 was flowed over immobilized gp140_CN54_ and revealed rapid binding with a slow dissociation rate (Figure 7A). To probe binding specificity we analyzed interference by PRO 2000 with the binding of mAbs of known epitope specificity to gp140 (Figure 7B). MAbs specific for the CD4i surface (E51, X5) and the V3 loop (19b) were potently inhibited, coordinate with the concept that polyanions bind these surfaces [18], [19]. By contrast, the epitope of the broadly neutralizing mAb b12 was not obscured by PRO 2000 but became modestly (∼40%) more exposed after PRO 2000 binding (Figure 7B) which may favour induction of antibodies of this specificity.

Since most proteases recognize sequences containing basic amino acids, and since PRO 2000 binds positively charged surfaces, we hypothesize that PRO 2000 might mask proteolytic cleavage sites and protect the antigen from degradation. To test the hypothesis we monitored antigen degradation in human vaginal lavage by Western blot (Figure 7C–D). In the absence of PRO 2000, gp140 was rapidly degraded into peptide fragments with a half-life of about 2 h, whereas in the presence of PRO 2000 the half-life was significantly extended to approximately 24 h. Thus PRO 2000 reversibly interacts with antigen protecting it from proteolytic degradation, thereby potentially increasing its residency time at mucosal surfaces.

## Discussion

The small animal vaginal immunization models described here provide proof-of-principle for two important concepts: 1) that vaginally-applied PRO 2000, a gel that is well-tolerated in a human vaginal context, can act as a B cell adjuvant to increase and sustain both local and systemic specific antibody responses; 2) that PRO 2000 is a TLR4 antagonist that reduces mucosal pro-inflammatory responses and so drives a Th2 immune bias. Further observations of potential benefit relate to the selective antigenic masking of gp140 epitopes that may lead to enhanced generation of more conserved antibody responses and protection of gp140 from proteolytic degradation leading to increased mucosal residency time.

The finding that gp140 formulated in PRO 2000 elicited specific mucosal and systemic B and T cell responses was encouraging since the vaginal mucosa has been considered a difficult site for induction of adaptive immunity [32], [33]. However, a more recent analysis has suggested that the rabbit vaginal vault may be an immune inductive site when very high doses of antigen are applied on a frequent basis [44]. Although our experiments were not specifically designed to evaluate the duration of the mucosal immune response to vaginal immunization, we nevertheless observed that mice immunized with antigen in PRO 2000 maintain specific IgA responses for up to 150 days after the last boost. Despite the relatively modest titres, this is promising as it suggests induction of long-lived local plasma cells that escape tolerogenic suppressive mechanisms. In rabbits, we observed the induction of equivalent IgA responses in presence or absence of PRO 2000 but significantly increased vaginal and systemic IgG in animals receiving gp140 in PRO 2000. This confirms the immunostimulatory activity of PRO 2000 in a second species, but indicates that the type of antibody response is species-specific. The observation that specific serum IgG and vaginal IgG titres correlate with each other and with the pseudovirus neutralizing capacity of vaginal lavage samples supports the notion that vaginal IgG is primarily derived from serum transudate [45], [46]. The neutralizing antibody titres demonstrated here were against a heterologous clade C Env-pseudotyped virus, demonstrating some breadth of activity. We acknowledge that titres were low and this pseudovirus Env is unusually sensitive to neutralization [47], but this result should encourage further development of this immunization concept with the use of next-generation Env-based antigens as they become available. Induction of antigen-specific IgG and IgA at the vaginal mucosa was modest, but encouragingly intranasal priming with experimental adjuvant prior to boosting with gp140-PRO 2000 further increased antibody responses. Although the mucosal antigen-specific antibody responses that we observe are of relatively low titre, they may be further optimized by increasing antigen dose, increasing the number of boosts, or adding a heterologous adjuvant prime as demonstrated in figure 3. Moreover, a recent study demonstrated that lower than previously suggested levels of neutralizing antibody may be sufficient to reduce transmission in a low-dose challenge model, and that antibody effector functions may play a role distinct from, or additional to, neutralization [48].

The finding that PRO 2000 drives an anti-inflammatory Th2-biased environment is of interest because of the potential risks of local inflammation resulting in increased susceptibility to HIV-1 infection. A pro-inflammatory environment may lead to local tissue damage, and may result in recruitment of HIV-1-susceptible target cells to the mucosae [11]. Furthermore, CCL2 may increase HIV-1 entry into resting CD4^+^ T cells [41] and triggers recruitment of HIV-1-susceptible target cells [42]. Thus reduced CCL2-secretion might defavour HIV-1 infection of the vaginal mucosa *in vivo*. We hypothesized that PRO 2000 might shift the immune response towards a Th2-type by inhibiting the local Th1 homeostasis probably induced by bacterial microflora. Indeed we found that PRO 2000 competitively inhibited the interaction between bacterial LPS and its cognate receptor TLR4, a potent inducer of Th1-type immune responses. Interestingly, PRO 2000 has recently been shown to block the LPS-induced cytokine response of human dendritic cells [49], which supports our finding. This result is consistent with the cellular cytokine profile we found in mice immunized in the presence of PRO 2000, since TNF-α and IL-1α are produced after LPS stimulation. The probable source of vaginal LPS is the local bacterial flora. Accordingly, lavage from women with bacterial vaginosis, an imbalance of naturally occurring bacterial flora, showed increased TNF-α secretion and TLR4 mRNA expression [50], illustrating the capacity of these flora to trigger immune responses.

These results, together with the ability of PRO 2000 to preserve antigen integrity in a vaginal environment and previously-published evidence that vaginally-applied PRO 2000 is safe in clinical trials with no evidence for local toxicity and good vaginal retention [14], [15], [16], make a case for further exploration of PRO 2000 as a topical vaccine formulation agent. Although PRO 2000 has failed to prevent sexual HIV-1 transmission in a phase-III clinical trial [51], our data support further exploration of its potential use as a mucosal vaccine-formulating agent capable of promoting antigen-specific immune responses whilst inhibiting local inflammation. This vaginal vaccine concept might also provide an ideal platform for the incorporation of next-generation microbicides, such as antiretroviral drugs, which would combine potential long-term protection offered by frequently-boosted mucosal antibody responses with short-term protection offered by microbicide use.

## Methods

### Ethics statement

All animal studies were performed in accordance with UK national guidelines (Animals (Scientific Procedures) Act 1986) and were authorized by the UK Home Office and the Oxford local institutional ethical review board (Project license no. 30/2390).

### Antigen

Trimeric gp140 was derived from HIV-1_97CN54_, a chinese clade B/C recombinant, CCR-5 tropic (R5) isolate (Accession no. AX149771). The near full-length clone p97CN54 was obtained originally from HIV isolated from a Chinese patient [52], [53] and was made available by H. Wolf and R. Wagner, University of Regensburg, Germany. Trimeric clade C gp140 (gp140_CN54_) was produced as a recombinant product in CHO cells and manufactured to GMP specification by Polymun Scientific, Vienna, Austria.

### Animals and immunizations

6–12 weeks old female BALB/c mice were used for the mouse immunogenicity studies. Intranasal immunizations were adjuvanted with polycation (Sigma-Aldrich, Gillingham, UK). Intravaginal immunizations were carried out by injecting 20 µl of antigen and/or 1% PRO 2000 (Indevus, now Endo Pharmaceuticals, Newark, DE) formulated in carrier gel into the vagina of the mice. The murine oestrous cycle was synchronized by injecting 1.5 mg of water-soluble progesterone (Sigma-Aldrich) per mouse three days prior to vaginal immunization. For subcutaneous immunizations, 0.1 ml of a 1∶1 mixture of antigen in PBS and FCA (Sigma-Aldrich) was injected into the flank of the mice. A subsequent subcutaneous boost immunization was performed without adjuvant in week 3 and serum samples were collected in week 5. Vaginal lavage samples were collected not sooner than 1 week after vaginal immunization by pipetting 2×50 µl of sterile PBS in and out of the vagina several times using sterile blunted micropipette tips.

For rabbit experiments, 8–10 weeks old female New Zealand Whites were used. All rabbits received a total of six intavaginal immunizations with 200 µl 5% hydroxyethylcellulose (HEC) gel containing 50 µg gp140 and/or 1% PRO 2000. Groups of 3 rabbits per experimental arm received an intranasal prime immunization in week 0 with 500 µg poly(I:C) and 50 µg gp140_CN54_ in 100 µl saline or with 500 µg poly(I:C) in 100 µl saline alone. Antibody titres between these subgroups were not statistically different at any time point of the experiment, therefore the intranasal prime was assumed to be ineffective in rabbits and results were pooled. Vaginal lavage samples were collected one week after vaginal immunization by injecting and withdrawing 0.5 ml of sterile PBS several times using a sterile catheter. The optimal gp140_CN54_ antigen dose to differentiate between adjuvanted versus non-adjuvanted mucosal immunization was determined empirically in previous experiments.

### Neutralization assays

Neutralization of clade C MW965 pseudovirus was assessed in a modified pseudovirus neutralization assay described previously [54]. Rabbit vaginal lavage samples collected prior to immunization (pre-immune samples) and week 12 after 6 intravaginal immunizations were tested at 1∶8 dilution. The mean infection level observed with pre-immune samples was used as the 100% infection level, and the relative neutralization of week 12 vaginal lavage samples was calculated accordingly. Neutralization was determined in duplicate in at least two independent assays.

### Stability of gp140 in human vaginal lavage (HVL)

HVL was collected with a sterile pipette, diluted 1∶1 in H_2_O and was stored at −80°C after insoluble components were removed by centrifugation. For the stability assay, 0.5 µg gp140_CN54_ was incubated in HVL in the presence or absence of 100 µM PRO 2000 at 37°C and stored at −80°C until analyzed. Samples were subjected to SDS-PAGE and analyzed by Western blotting.

### Sample preparation and ELISA

Vaginal lavage and serum samples were cleared by centrifugation at 20,000× g at 4°C and stored at −20°C until analyzed. Antigen-specific and total antibody levels were determined by ELISA. High protein binding ELISA plates were coated with gp140_CN54_ in PBS, blocked with 2% skimmed milk powder in washing buffer (WB, 0.05% Tween-20 in PBS) for mouse experiments, 2% BSA in WB for rabbit IgG, 10% goat serum in WB for rabbit IgA and incubated with serial dilutions of samples. For rabbit total IgG and IgA assays, plates were coated with capture antibody (Serotec 403001, Abcam ab2758, respectively) and blocked with 10% goat serum in WB. Bound antibodies were detected with the appropriate secondary reagents: anti-mouse IgG-HRP (STAR120P, Serotec, Oxford, UK); anti-mouse IgA (STAR85, Serotec) conjugated to biotin; anti-mouse IgG1-HRP and IgG2a-HRP (BD Biosciences, Oxford, UK); anti-rabbit IgG-HRP (Sigma-Aldrich); anti-rabbit IgA (A120-109A, Bethyl, Montgomery, TX) conjugated to biotin; TMB substrate (Thermo Fisher Scientific, Rockford, IL); and OD values were read at 450 nm.

Competition ELISA was performed using immobilized gp140_CN54_ and binding of epitope-defined human mAbs was detected using HRP-conjugated goat anti-human IgG Fcγ. mAb specific for the CD4-induced site were co-incubated with 2 µg/ml sCD4.

ELISA data were analyzed by subtracting the plate background from all readings and by fitting a sigmoidal dose response curve to the data for each sample by using the Prism software (Graphpad, San Diego, CA). The endpoint titre was determined from the maximum dilution of sample at which the OD_450_ signal was greater than the threshold (0.01), which was always greater than two standard deviations above background.

### Culture of inguinal lymph node cells and cytokine assay

One week prior to the isolation of cells, all mice described in Figure 3A–C received a final vaginal immunization with the same formulations as before. Cells derived from the vagina draining inguinal lymph nodes of these mice were isolated and culfotured for three days in the presence or absence of 15 µg/mL gp140. Cytokine profiling was done by Bio-Plex cytokine assay (Bio-Rad, Hercules, CA) according to the manufacturers instructions.

### TLR reporter cell assay

Immortalized mouse embryonic fibroblasts (C3H/WT MEF, Invivogen, San Diego, CA) were used as TLR-reporter cell line. Briefly, cells were seeded on day 0 at 1×10^4^ per well in 96-well plates and cultured overnight. On day 1, 130 µL medium was replaced with medium containing the appropriate concentrations of TLR-agonist (Invivogen) and/or PRO 2000. On day 2, 20 µL of culture supernatant was removed and the phosphatase content was quantified by colorimetric substrate reaction (P5994, Sigma-Aldrich).

### Surface plasmon resonance (SPR)

SPR analysis was performed on a BIAcore 2000 biosensor (GE-Healthcare) by coupling gp140_IIIB_ and gp140_CN54_ to a CM5 sensor chip to produce 11000-12000 RU. The antigenicity of gp140 was analyzed using different HIV-1 Env-specific mAbs (50 µg/mL), sCD4 (25 µg/mL) or combinations thereof. Recombinant human TLR4-MD2 complexes (R&D Systems, Minneapolis, MN) were immobilized on a CM5 sensor chip at approximately 8000 RU. The immobilization of lipid A (Sigma-Aldrich) onto the HPA biosensor chip was carried out as described previously [55]. Samples were injected for 2 min at a flow rate of 20 µL/min.

### Total Ig determination by multiplex immunoassay

The total IgG and IgA concentration of mouse vaginal lavage was determined using a multiplex mouse immunoglobulin isotyping kit (Millipore, Billerica, MA) following the manufacturer's instructions. Assays were read on a Bio-Plex system (Bio-Rad). Total IgG and IgA concentrations in µg/ml were used to normalize mucosal IgG and IgA endpoint titres.

### Blue native PAGE

Blue native PAGE was performed essentially as described elsewhere [56]. Briefly, proteins were diluted in 50 mM Bis-Tris buffer pH 7.0 and were separated on 3-12% Bis-Tris gels (Invitrogen, Paisley, UK) in the presence of a final concentration of 0.05% Triton x-100. After electrophoresis, gels were silver-stained to visualize less abundant protein fractions.

### Statistical analysis

Endpoint titres were log-transformed and analyzed using a Kolmogorov-Smirnov normality test. If the data showed a normal distribution within each compared group an unpaired *t*-test with a confidence interval of 95% was used to assess for statistical significance defined as *p*<0.05. If the data were not normally distributed, they were analyzed using a Mann-Whitney test with the same confidence interval and significance limit. For antibody titres, results are presented as geometric group means or were log-transformed and presented as means ± SEM; all other data are presented as means ± SD.

## Supporting Information

Figure S1 **Biochemical characterization of the immunogen.** (**A**) Antigenic analysis of HIV-1 gp140_CN54_ compared to HIV-1 IIIB gp140 using a panel of epitope-defined mAbs and soluble CD4. The relative binding of the analytes in a surface plasmon resonance based binding assay is given in response unit (RU)-ranges: -  =  0-150RU; +  = 151-500 RU; ++  = 501-1500 RU; +++  =  above 1501 RU. Means of two independent experiments are shown (**B**) Blue native PAGE analysis of the antigen. gp140_CN54_ shows an apparent native molecular weight of approximately 480 kDa.
(TIF)

Figure S2 **Total Immunoglobulin content of vaginal lavage samples and correlation of rabbit antigen-specific vaginal- and serum IgG.** Total rabbit IgA (**A**) and IgG (**B**) content of vaginal lavage samples and Pearson correlation (**C**) of week 11 rabbit vaginal and serum IgG responses from the experiment depicted in Figure 2.
(TIF)
