# Supplementary figures and images for: A Novel Strategy for Inducing Enhanced Mucosal HIV-1 Antibody Responses in an Anti-Inflammatory Environment
Source: PLoS One. 2011 Jan 6;6(1):e15861. doi: 10.1371/journal.pone.0015861 (PMC3017049; doi:10.1371/journal.pone.0015861)

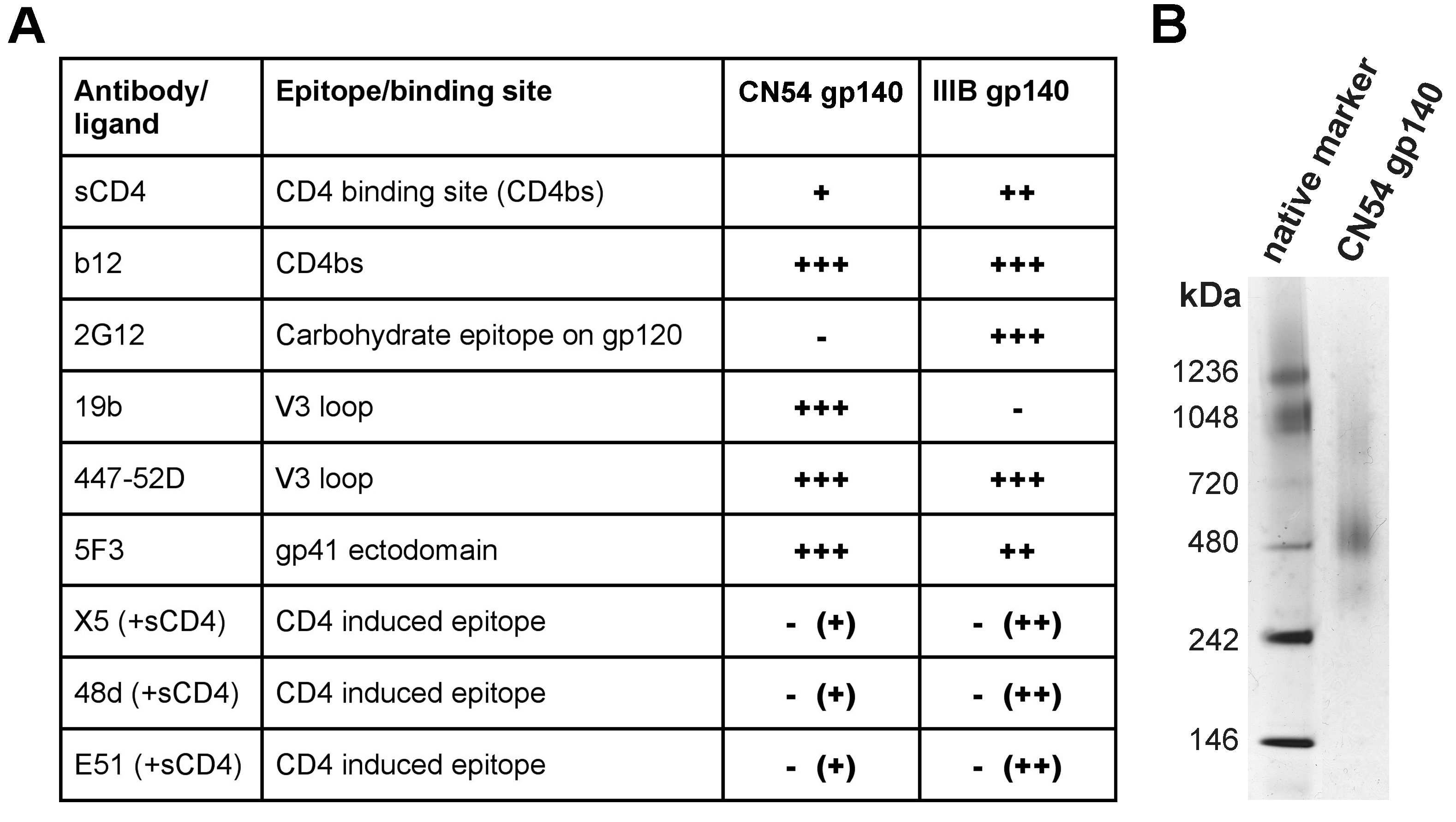

Supplement: Figure S1 — Biochemical characterization of the immunogen. (A) Antigenic analysis of HIV-1 gp140CN54 compared to HIV-1 IIIB gp140 using a panel of epitope-defined mAbs and soluble CD4. The relative binding of the analytes in a surface plasmon resonance based binding assay is given in response unit (RU)-ranges: - = 0-150RU; + = 151-500 RU; ++ = 501-1500 RU; +++ = above 1501 RU. Means of two independent experiments are shown (B) Blue native PAGE analysis of the antigen. gp140CN54 shows an apparent native molecular weight of approximately 480 kDa. (TIF) [file pone.0015861.s001.tif]

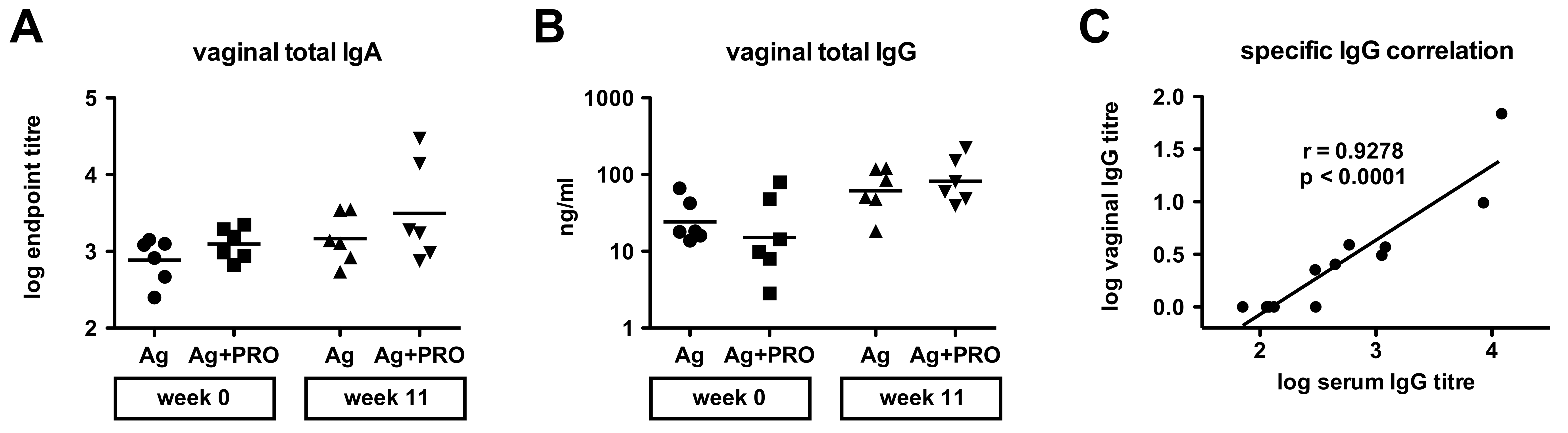

Supplement: Figure S2 — Total Immunoglobulin content of vaginal lavage samples and correlation of rabbit antigen-specific vaginal- and serum IgG. Total rabbit IgA (A) and IgG (B) content of vaginal lavage samples and Pearson correlation (C) of week 11 rabbit vaginal and serum IgG responses from the experiment depicted in Figure 2. (TIF) [file pone.0015861.s002.tif]
